# Supplementary material for: Nutritional and Functional Advantages of the Use of Fermented Black Chickpea Flour for Semolina-Pasta Fortification
Source: Foods. 2021 Jan 18;10(1):182. doi: 10.3390/foods10010182 (PMC7831118; doi:10.3390/foods10010182)
Supplement: Supplementary file 1 [file foods-10-00182-s001.pdf]

foods

MDPI

Supplementary material - Nutritional and Functional Advantages of the Use of Fermented Black Chickpea Flour for Semolina-Pasta Fortification.

Ilaria De Pasquale, Michela Verni, Vito Verardo, Ana María Gómez-Caravaca and Carlo Giuseppe Rizzello

Table 1. Drying cycle used for making pasta.

	Ventilation			Recovery		
Cycle	Temperature (°C)	U.R.%	Time (min)	Temperature (°C)	U.R.%	Time (min)
1	55	85	3	55	85	2
2	55	85	3	55	85	2
3	55	70	3	55	70	4
4	55	70	3	40	65	8
5	55	70	3	40	60	10
6	55	70	3	40	60	10
7	55	70	3	40	55	10
8	55	70	3	40	55	12
9	55	70	3	40	55	12
10	55	70	3	40	55	12
11	55	70	3	40	55	12
12	55	70	3	40	55	14
13	55	70	3	40	50	14
14	55	70	3	40	50	16
15	55	70	3	40	50	16
16	55	70	3	40	50	16
17	55	65	3	40	50	16
18	55	65	3	40	50	18
19	55	65	3	40	50	18
20	55	65	3	40	45	18
21	55	65	3	40	45	18
22	55	60	3	35	45	18
23	55	60	3	35	45	18
24	50	55	3	35	45	18
25	45	55	3	35	15	18
26	40	40	3	15	15	10
27	15	10	20	15	10	20
28	15	10	20	15	10	20

Table 2. List and definition of the attributes used for the sensory analysis made on pasta samples.

Attribute	Abbreviation	Definition
		Visual
Color heterogeneity	Ht	Heterogeneity of the pasta color evaluated after cooking
Texture	Te	Uniformity of the pasta texture at visual inspection
Stacking	St	The degree of piece to piece adhesion
		Odor
Intensity	OI	Overall odor intensity of pasta
Pungent	OP	Pungency of pasta smell
Flavour		
Pungent	P	Pungency of pasta flavour
Delicate	D	Soft and delicate flavor perception
Legume	L	Typical flavor of lentils, beans, chickpeas, etc.
Whole	W	Flavor of whole-wheat flour, buckwheat
Toasted	To	Grilled and toasty flavor
Sapidity	Sp	Sapidity taste
		Texture
Chewability	Ch	The mouthfeel sensation of labored mastication due to sustained, elastic resistance from the sample.
General acceptability	GA	Overall appreciation

Table 3. Polar compounds identified in free and bound extracts of black chickpea doughs by UPLC-PDA-ESI-QTOF.

No.	Polar compound	RT (min)	Molecular Formula	m/z expected	m/z calculated	Error (ppm)	Score (%)	Fragments (MS[2])
Free phenolic compounds								
**1**	Protocatechuic acid	1.125	$C_7H_6O_4$	153.0198	153.0188	6.5	100	
**2**	Dihydroxybenzoic acid hexoside	2.652	$C_{13}H_{16}O_9$	315.0719	315.0716	1	96.95	217.0164, 152.0088, 108.0225
**3**	Dihydrocaffeic acid	2.921	$C_9H_{10}O_4$	181.0512	181.0501	6.1	92.63	
**4**	Indole-3-acrylic acid	3.376	$C_{11}H_9N_1O_2$	186.0568	186.0555	7	89.47	142.0665
**5**	Hydroxybenzoic acid hexoside-pentoside	3.521	$C_{18}H_{24}O_{12}$	431.1194	431.119	0.9	99.95	137.0245
**6**	Dihydroxybenzoic acid hexoside-pentoside I	4.030	$C_{18}H_{24}O_{13}$	447.1144	447.1139	1.1	96.67	131.0708
**7**	Dihydroxybenzoic acid hexoside-pentoside II	4.336	$C_{18}H_{24}O_{13}$	447.1144	447.1139	1.1	96.67	131.0708
**8**	Dihydrophaseic acid	4.518	$C_{15}H_{22}O_5$	281.1394	281.1389	1.8	96.55	217.004
**9**	Phloretic acid	5.54	$C_9H_{10}O_3$	165.0564	165.0552	7.3	62.13	
**10**	Dihydrophaseic acid isomer	6.165	$C_{15}H_{22}O_5$	281.1394	281.1389	1.8	96.55	217.0029
**11**	Hydroxybenzoic acid hexoside-pentoside dehydrodimer	6.634	$C_{36}H_{46}O_{23}$	845.2342	845.2352	−1.2	99.99	431.1199, 137.0250
**12**	Hydroxybenzoic acid hexoside-pentoside trimer	7.753	$C_{54}H_{68}O_{34}$	1259.3509	1259.3514	−0.3	77.69	845.2329, 431.1147, 137.0228
**13**	Hydroxibenzoic acid derivative	8.390	$C_{25}H_{28}O_{14}$	551.1398	551.1401	−0.5	99.01	413.1053, 137.0256

**14**	Myricetin derivative	9.154	$C_{32}H_{38}O_{21}$	757.1796	757.1827	−4.1	43.77	316.0228
**15**	Quercetin-3-7-O-di-glucopyranoside	9.342	$C_{27}H_{30}O_{17}$	625.1426	625.1405	3.4	95.9	
**16**	Quercetin-3-O-β-D-xylopyranosyl-(1/2)-rutinoside	10.018	$C_{32}H_{38}O_{20}$	741.1866	741.1855	−1.6	92.81	300.0274
**17**	Myricetin-O-methyl ether hexoside deoxy-hexoside pentoside	10.064	$C_{33}H_{40}O_{21}$	771.1972	771.1984	−1.6	99.98	315.015
**18**	Kaempferol 3-O-lathyroside-7-O-α-L-rhamnopyranoside	10.699	$C_{32}H_{38}O_{19}$	725.1915	725.1929	−1.9	99.97	284.0333
**19**	Quercetin-3-O-rutinoside-7-O-α-L-rhamnopyranoside	10.823	$C_{33}H_{40}O_{20}$	755.2009	755.2035	−3.4	99.56	299.0200, 300.0274
**20**	Lablab saponin	16.771	$C_{54}H_{82}O_{22}$	1081.5216	1081.5219	−0.3	99.93	
**21**	Soyasaponin Bd	17.206	$C_{48}H_{77}O_{18}$	941.5102	941.511	−0.8	96.95	
Bound phenolic compounds								
**1**	Gallic acid	1.275	$C_7H_6O_5$	169.0147	169.0137	1	99.28	125.0243
**2**	Protocatechuic acid	1.685	$C_7H_6O_4$	153.0198	153.0188	6.5	100	
**3**	Dihydroxybenzoic acid hexoside	2.131	$C_{13}H_{16}O_9$	315.0721	315.0716	1.6	99.56	217.0038, 152.0105, 108.0221
**4**	Hydroxybenzoic acid hexoside-pentoside	3.463	$C_{18}H_{24}O_{12}$	431.1195	431.119	1.2	82.5	
**5**	Morin	3.956	$C_{15}H_{10}O_7$	301.0362	301.0402	−13.3	99.08	
**6**	Dihydroxybenzoic acid hexoside-pentoside I	4.001	$C_{18}H_{24}O_{13}$	447.1125	4471139	−3.1	78.66	
**7**	Dihydrophaseic acid	6.120	$C_{15}H_{22}O_5$	281.1384	281.1389	−1.8	95.61	217.0029

**8**	Ferulic acid	7.642	$C_{10}H_{10}O_4$	193.0494	193.0501	−3.6	62.23	
**9**	Isoferulic acid	8.291	$C_{10}H_{10}O_4$	193.0504	193.0501	1.6	84.77	
**10**	Myricetin-O-methyl ether hexoside deoxy-hexoside-pentoside	10.028	$C_{33}H_{40}O_{21}$	771.1976	771.1984	-1	59.56	315.015
**11**	Kaempferol 3-O-lathyroside-7-O-α-L-rhamnopyranoside	10.645	$C_{32}H_{38}O_{19}$	725.1946	725.1929	2.3	99.31	284.0333
**12**	Soyasaponin Ba	16.031	$C_{48}H_{76}O_{19}$	955.4886	955.4903	−1.8	49.05	
**13**	Soyasaponin Bd	17.190	$C_{48}H_{78}O_{18}$	941.5096	941.5110	−1.5	90.4	
